# Effects of Process Parameters on Defect Formation in Laser Additive Manufacturing of a Novel Ni-Based Superalloy

**DOI:** 10.3390/ma18133102

**Published:** 2025-07-01

**Authors:** Wen-Tao Liu, Jing-Cheng Zhou, Jing-Jing Ruan, Hua Zhang, Xin Zhou, Liang Jiang, Li-Long Zhu

**Affiliations:** 1 School of Materials Science and Engineering, Shandong University of Science and Technology, Qingdao 266590, China; liuwentao314@163.com; 2 Institute for Advanced Studies in Precision Materials, Yantai University, Yantai 264005, China; jingchengcz@s.ytu.edu.cn (J.-C.Z.); ruanjingjingtohoku@163.com (J.-J.R.); zhanghua@ytu.edu.cn (H.Z.); zhouxin@ytu.edu.cn (X.Z.); 3 Shandong Key Laboratory of Advanced Structural Materials Genome Engineering, Yantai University, Yantai 264005, China

**Keywords:** laser-directed energy deposition, Ni-based superalloy, process parameters, defects, cracking mechanisms

## Abstract

Laser additive manufacturing offers significant advantages for fabricating and repairing complex components. However, the complex solidification and remelting processes in nickel-based superalloys for additive manufacturing can introduce defects such as voids and cracks. Therefore, process parameters are crucial, as they significantly impact solidification and remelting, thereby affecting defect formation. In this study, laser-directed energy deposition was employed to evaluate the effects of our key process parameters on the formation of voids and cracks in a novel superalloy. The findings reveal that laser power and linear energy density significantly influence the void content and crack density. However, the influence of other process parameters on defect formation is relatively minimal. The optimal parameter space is characterized by a laser power range of 600~700 W, a linear energy density range of 60~90 J/mm and a powder feeding rate of 0.7~0.8 rpm. Moreover, the precipitation of fine MC-type carbides near the dendrites and grain-boundary misorientations within the range of 31~42° are associated with a higher propensity for crack formation. These insights provide a valuable reference for controlling the process parameters and understanding the cracking mechanisms in laser additive manufacturing of superalloys.

## 1. Introduction

Ni-based superalloys are essential structural materials for the key thermal components of turbine engines due to their exceptional properties at high temperatures, including high strength and excellent resistance to creep, fatigue, corrosion and oxidation [1,2,3]. Critical components such as turbine discs and blades typically have highly intricate geometries. The machining of these components using traditional processes is not only complex but also compromises the mechanical properties of the material due to the required welding. Laser additive manufacturing (AM) technology, on the other hand, enables the direct printing of alloy powders in complex structures, thereby demonstrating significant potential to overcome these limitations [4]. The AM of complex superalloy components enables direct formation, significantly reducing processing time and material waste [5,6]. The integrity and mechanical properties of the superalloy components are also improved by eliminating the welding process.

Various advanced Ni-based superalloys, such as CM247LC [7,8], Hastelloy X [9,10], IN718 [11,12], IN625 [13,14], IN738 [15,16], IN738LC [17,18], IN939 [7,19], SRR99 [20], Haynes282 [21], AD730 [22], ABD-900AM [7], ABD-850AM [7] and CMSX-4 [23], have been successfully produced using AM techniques. However, due to the high crack susceptibility of Ni-based superalloys, inappropriate employment of parameters during the AM process can lead to numerous defects, including voids and microcracks [24,25,26]. These defects can significantly compromise the mechanical properties and performance of the manufactured components. Therefore, devising strategies to mitigate defects in the AM of Ni-based superalloys remains a crucial aspect.

As with traditional processing methods of casting and welding, AM parts undergo rapid and repeated heating and cooling cycles during the layer-by-layer deposition process of laser-directed energy deposition (L-DED). This cyclical thermal exposure can lead to the formation of unique microstructural characteristics and defects. Porosity and cracking are two of the most common concerns in metal AM [27]. Porosity is a pore-like defect and can be mainly categorized as gas pores, keyhole pores and lack-of-fusion pores [28,29]. Gas pores are spherical and relatively small, primarily caused by gas trapped in raw metal powders or protective gas entrapped during the fabrication process. Keyhole pores occur due to excessive energy input during the melting process [30]. High beam energy causes excessive penetration of metal powders, resulting in deep and narrow pores near the bottom of the melt pool after solidification. Conversely, insufficient energy input leads to lack-of-fusion pores [31,32]. In this case, the lower energy level fails to fully melt the metal powders, leaving relatively large and irregularly shaped pores. In addition to pores, well-aligned chains of rounded voids, referred to as micro-shrinkage, are also observed along the direction of columnar grain growth in the AM parts, each typically having an average diameter of less than 5 μm. Chauvet et al. [32] proposed that these structures originate from the coalescence of secondary arms of intragranular dendrites during the final stage of solidification.

Cracking is a critical issue for AM parts, typically occurring during the solidification or subsequent heating processes. The primary mechanisms of cracking can be categorized as solidification cracking, liquation cracking, strain age cracking and ductility dip cracking [7,33,34]. Among these, solidification cracking and liquation cracking involve the appearance of liquid film, often due to strong thermal gradients during rapid solidification and the formation of low-melting-temperature regions that can initiate cracks. In contrast, ductility-dip cracking and strain-age cracking occur in the solid state. Ductility-dip cracking happens when the ductility of alloys significantly decreases within a specific temperature range (typically 0.7 to 0.5 times the melting point of the alloy), leading to intergranular cracking under thermal stress. Strain-age cracking, on the other hand, arises from the combination of residual stresses and the precipitation of brittle phases during post-aging heat treatment, which embrittle the alloy and make it susceptible to cracking. Key factors contributing to cracking primarily include the temperature gradient (TG) [35], high-angle grain boundaries (HAGBs) [33,36], interdendritic precipitation of carbides [37] and low-melting-point (γ + γ′) eutectics [38]. Several process parameters in AM, such as laser power, scanning speed, linear energy density, powder feeding rate and the width of the clad track (including laser preheating and temperature variation), play a vital role in determining the microstructure and physical/mechanical properties of the manufactured parts [39,40,41,42]. These parameters are interdependent; specifically, the linear energy density, which is crucial for material fusion and heat distribution, is determined by the ratio of laser power to scanning speed. Understanding and effectively controlling these interdependencies is essential for optimizing the manufacturing process to minimize defects and stress risers, thereby achieving the desired microstructure and mechanical properties in the final components.

In the present work, we investigated the influence of process parameters on defect formation in a novel Ni-based superalloy during L-DED. A series of 25 bulk samples were prepared under varying conditions, and their microstructures were systematically examined to identify optimal process parameters. Furthermore, the impact mechanisms of these parameters on defect formation were thoroughly analyzed.

## 2. Materials and Methods

### 2.1. Characterization of Ni-Based Superalloy Powders

Ni-based superalloy powders with a nominal particle size distribution (PSD) ranging from 45 to 105 μm were produced by vacuum induction melting inert gas atomization (VIGA). The main chemical compositions of the powders were measured and reported in Table 1. The actual PSD and the sphericity were measured by means of a CAMSIZER X2dynamic image analysis system (Retsch GmbH, Haan, Germany). The crystal structure of Ni-based superalloy powders was obtained using a MiniFlex 600 X-ray diffractometer (XRD) (Rigaku Holdings Corporation, Tokyo, Japan) with Cu-Kα radiation operating at 40 kV and 40 mA.

### 2.2. L-DED Fabrication of Ni-Based Superalloy

**Figure 1:**
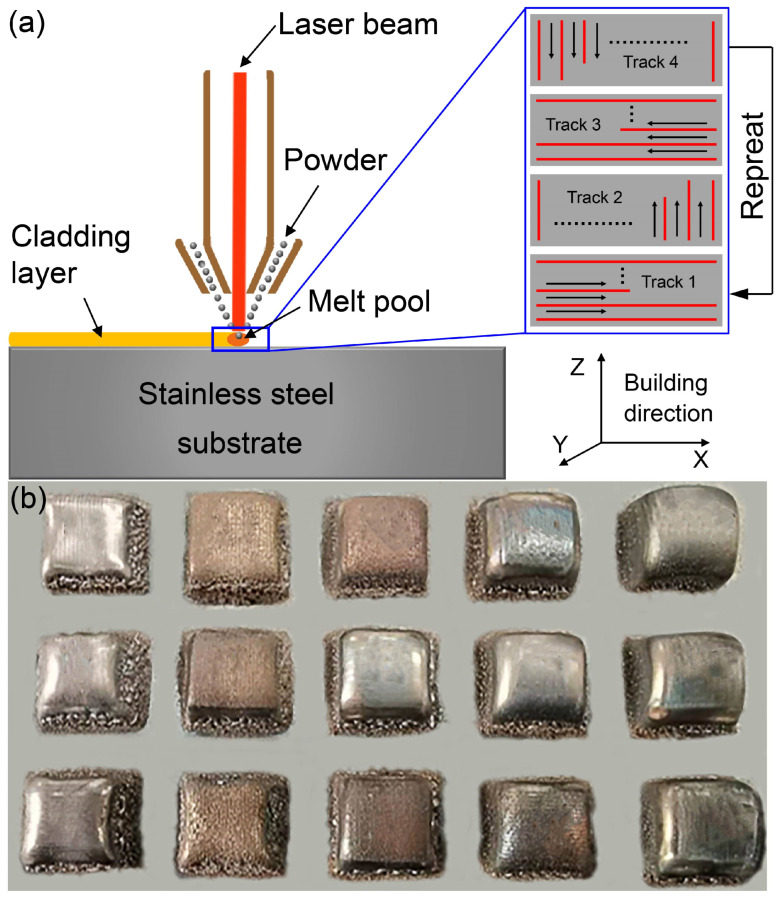
(**a**) Schematic illustration of the L-DED system and scanning paths during laser cladding; (**b**) photograph of a portion of the L-DED Ni-based superalloys.

The Ni-based superalloy samples were fabricated using a iLAM^®^511C L-DED system (Nanjing Huirui Photoelectric Technology Co., Ltd., Nanjing, China) employing a 1070 nm wavelength laser with a maximum power output of 1 kW. The Ni-based superalloy powder streams were carried by high-purity argon through three gas lines to a coaxial powder feeding nozzle, where they were sprayed out, then merged to form a focused powder stream aligned with the center axis of the laser beam. In this study, the powder feeding system continuously dispensed 8.06 g of Al superalloy powder per revolution, ensuring a precise and consistent material flow throughout the cladding process. Eventually, the Ni-based powders were fabricated into cubic specimens, each with dimensions of approximately 15 × 15 × 15 mm^3^, on a stainless-steel substrate. The entire manufacturing process was conducted in an argon-filled glovebox with oxygen and water levels maintained below 10 ppm and 0.01 ppm, respectively. The L-DED process, along with the laser scanning paths, is schematically illustrated in Figure 1a. An X/Y orthogonal scanning strategy was employed between each successive layer to achieve more homogeneous melting, ensuring consistent overlap and even distribution of the cladded superalloy. As shown in Figure 1a, the cladding approach was implemented as Track 1 for the 1st, 5th, 9th and subsequent deposited layers. Correspondingly, Tracks 2, 3 and 4 were assigned to the 2nd, 3rd and 4th or additional cladding layers, respectively.

As mentioned above, the laser power, scanning speed, powder feeding rate and hatching space are the four key process parameters for metal AM. Therefore, these critical variables were systematically employed in this investigation to evaluate their influence on the microstructural evolution of additively manufactured Ni-based superalloys, with corresponding experimental parameters detailed in Table 2. As a result, 25 samples were fabricated and thoroughly analyzed to produce a detailed and comprehensive dataset of results. To determine the energy delivered to the powders, one effective approach is the use of linear energy density (LED). For all conditions, the LED values were calculated using Equation (1) [14].

(1) $$E_{L}(J/mm)=Laser\;power\;(W)/Scan\;speed\;(mm/s)$$

### 2.3. Characterization of Ni-Based Superalloy

The as-built Ni-based superalloys were sectioned parallel to the building direction (*Z*-axis) using DK7732 wire electro-discharge machining (EDM) (Suzhou Bao Ma Numerical Control Equipment Co., Ltd., Suzhou, China). The cut surfaces of the samples were sequentially ground with SiC papers graded at 240, 400, 800, 1500 and 2000 grit, followed by gentle polishing using 1 μm and 0.3 μm Al_2_O_3_ solutions and concluded with vibratory polishing using 0.05 μm colloidal silica.

A M2700 M optical microscope (OM) (Leica Microsystems GmbH, Wetzlar, Germany) was employed to capture around 60 images of the XZ plane at a 100× magnification for each sample, covering a large region of approximately 17 mm^2^. ImageJ (v1-53c) software was utilized to seamlessly merge these images and perform a detailed analysis of the defect distribution and statistical characteristics. A manual threshold was set to individually count voids and cracks. Metrics including crack length/count density were employed to provide a detailed quantification of crack density. The microstructures and phase compositions of the samples were examined using a VEGA scanning electron microscope (SEM) (TESCAN ORSAY HOLDING, Brno, Czech Republic) that enables secondary electron (SE) and backscattered electron (BSE) imaging, as well as energy dispersive spectrometer (EDS) and electron backscattered diffraction (EBSD) measurements.

## 3. Results and Discussion

### 3.1. Ni-Based Superalloy Powders

**Figure 2:**
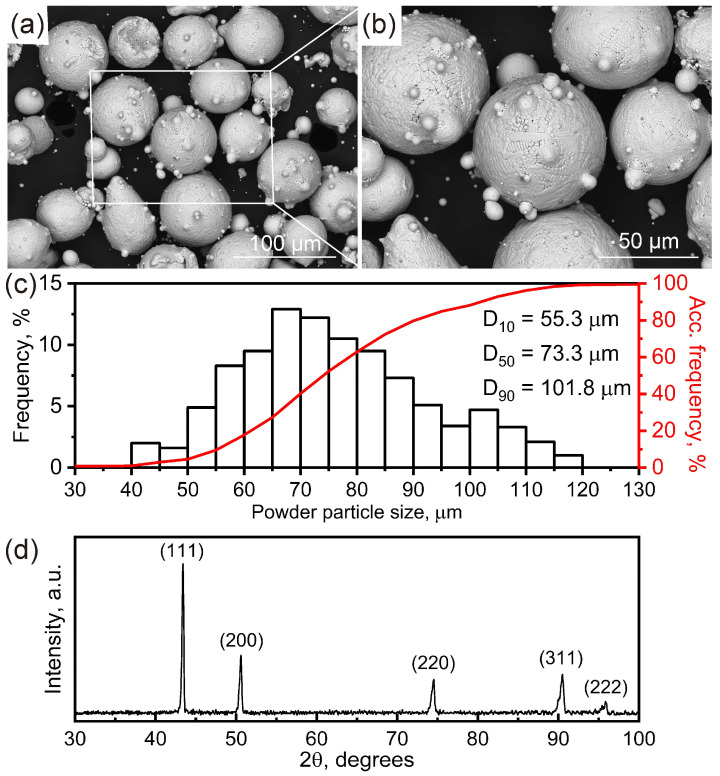
The measured characteristics of the Ni-based superalloy powders: (**a**) SE SEM image showing the powder morphology; (**b**) high-magnification image of the white box in (**a**); (**c**) histogram of the PSD; (**d**) XRD patterns identified for FCC γ-Ni according to Joint Committee on Powder Diffraction Standards (JCPDS) card No. 47-1417. The abbreviation “a.u.” in (**d**) denotes arbitrary units.

Figure 2a,b are secondary electron (SE) SEM images of the Ni-based superalloy powders, revealing a predominantly spherical particle morphology, with a few satellite attachments visible on the particle surfaces. Figure 2c presents the PSD of the Ni-based superalloy powders, demonstrating a typical Gaussian-type distribution. The measured D_10_ (10% of particles below this diameter), D_50_ (median particle size) and D_90_ (90% of particles below this diameter) values are 55.3 μm, 73.3 μm and 101.8 μm, respectively. These values align with the operational specifications of the employed L-DED apparatus. The measured average particle sphericity value is 77.6%, which is slightly below the 80% industry benchmark due to the presence of satellite particles. This deviation potentially increases the risk of crack initiation during additive manufacturing processes, particularly in powder bed fusion (PBF) systems, where flowability and packing density are critical. As confirmed by the XRD patterns in Figure 2d, the powder exhibits a face-centered cubic (FCC) γ-Ni matrix.

### 3.2. Quantitative Analysis of Defects in Alloy Microstructure

**Figure 3:**
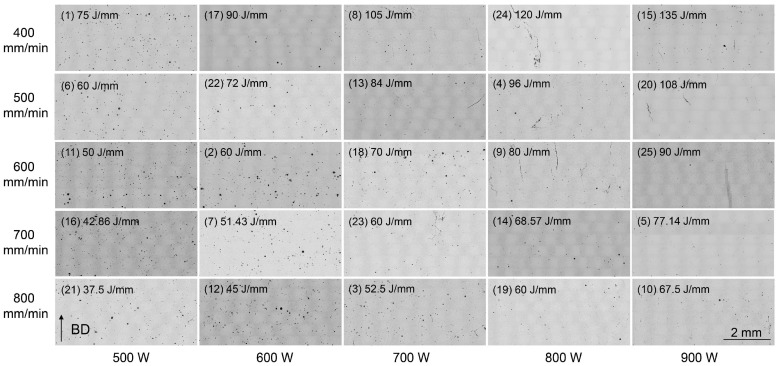
OM images displaying the microstructures of the Ni-based superalloys deposited with varying parameters.

OM imaging was initially employed to detect the presence of voids and/or cracks in the microstructure of the as-built Ni-based superalloy. Figure 3 displays the stitched OM images taken from the longitudinal XZ plane of the 25 cubic samples that were deposited using the corresponding parameters listed in Table 3. It is evident that voids and cracks are the two primary defects observed in the as-built Ni-based superalloy. Overall, voids are the predominant defects at relatively lower laser powers, while cracks become the main defect at higher laser powers, particularly when the scanning speeds are reduced. A detailed quantitative stereological analysis was conducted to accurately quantify both the void content and the crack density. The void content was determined by calculating the total area of voids as a percentage of the overall area of the stitched OM image. Both the crack count density and crack length density were adopted to facilitate a detailed analysis of crack distribution across each bulk sample [43]. The crack count density and crack length density were calculated using formulas of *n*/*A* and *L*/*A*, respectively. Here, *n* and *L* represent the total number of cracks and the total length of cracks (*L* = *L*_1_ + *L*_2_ + *L*_3_ + … + *L_n_*) within the statistical capture region, respectively, and A specifies the area of this region [39].

The statistical data on void content and crack density for all 25 bulk samples are comprehensively detailed and summarized in Table 3. Sample No. 16, fabricated using a line energy density of 42.86 J/mm and a laser power of 500 W, exhibits the highest void content of 1.23% while maintaining relatively low crack count and length densities of 0.77 pieces/mm^2^ and 0.134 mm/mm^2^, respectively. The lowest void content of 0.13% was confirmed in sample No. 24, accompanied by a relatively high crack length density of 0.35 mm/mm^2^. Sample No. 20, produced with a linear energy density of 108 J/mm and a laser power of 900 W, exhibits the highest crack count density of 2.08 pieces/mm^2^ and a crack length density of 0.55 mm/mm^2^ while maintaining a very low void content of 0.19%. No cracks were observed in sample Nos. 6 and 7, but they displayed relatively high void contents of 0.75% and 0.77%, respectively.

**Figure 4:**
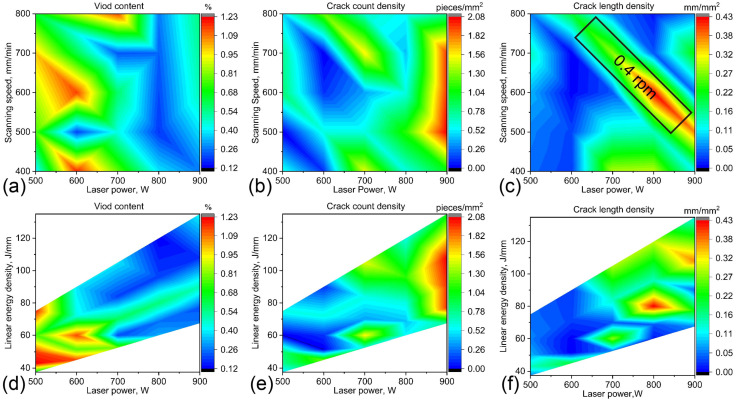
Comprehensive analysis of defect quantities across all 25 bulk samples: (**a**–**c**) void contents, crack count density and crack length density at varying laser powers and scanning speeds; (**d**–**f**) void contents, crack count density and crack length density at varying laser powers and linear energy densities.

Figure 4 illustrates a quantitative analysis of void content and crack density in 25 bulk samples, varying with distinct processing parameters utilized during fabrication. As evidenced in Figure 4a, void content appears to be relatively high at lower laser powers, yet it exhibits a noticeable decrease with increasing laser power. The void content increases slightly again at the highest power setting of 900 W. It can be seen in Figure 4b,c that both crack count density and crack length density are maintained at comparatively low levels within the approximate laser power range of 500 to 700 W. Laser power settings that exceed approximately 700 W correlate with an increase in crack density.

The laser scanning speed also affects the formation of voids and cracks, but its impact is generally less pronounced compared to that of laser power, as depicted in Figure 4a–c. Consequently, LED, calculated as the ratio of laser power to scanning speed according to Equation (1), was employed to evaluate the influence of the scanning speed on defect formation. Figure 4d–f illustrate variations in void content, crack count density and crack length density with respect to different laser powers and linear energy densities. It is observed that lower linear energy densities and laser powers tend to produce higher void contents, whereas higher linear energy densities and laser powers are associated with increased crack count and length densities. This variation trend has been verified across multiple alloy systems [44,45,46].

**Figure 5:**
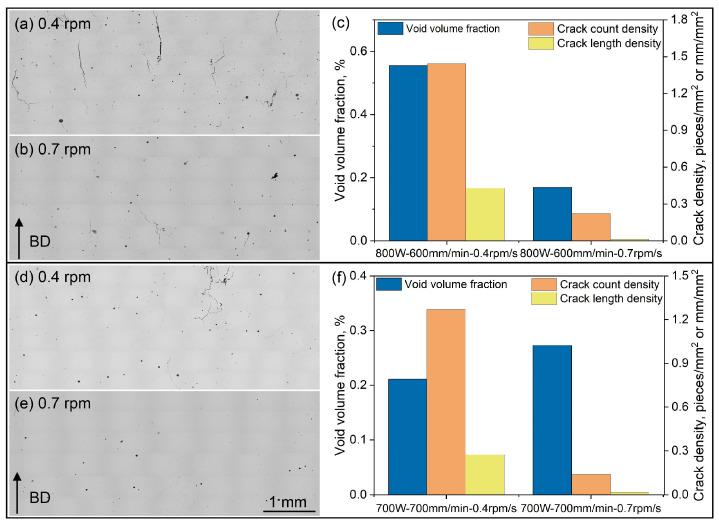
Defect analysis for the two samples conducted by increasing their powder feeding rate from 0.4 rpm to 0.7 rpm: (**a**–**c**) sample No. 9; (**d**–**f**) sample No. 23.

It is noteworthy that across the various laser power settings depicted in Figure 4e,f, there exists a distinct region where the crack length density significantly increases. This increase is particularly evident within the region highlighted by the black box in Figure 4c. All of these samples were produced using the lowest powder feeding rate of 0.4 rpm. The comparatively low powder feeding rate leads to an insufficient supply of the liquid phase for dendritic growth during the laser cladding process, which, in turn, results in a higher likelihood of solidification cracking [47]. Furthermore, the lower powder feeding rate leads to the formation of a thinner cladding layer, which concentrates a higher amount of laser energy within the unit volume of the sample. This increased energy density can induce thermal stress, thereby increasing the risk of solid-state cracking within the front cladding layer. A similar observation was also reported by Segerstark et al. [48] during their optimization of AM process parameters for IN718. To further validate these findings, the powder feeding rate for sample Nos. 9 and 23 was increased from 0.4 rpm to 0.7 rpm, with all the other process parameters remaining constant. As demonstrated in the defect analysis of Figure 5, this adjustment to a higher powder feeding rate led to significant decreases in both voids and cracking within the alloy samples.

**Figure 6:**
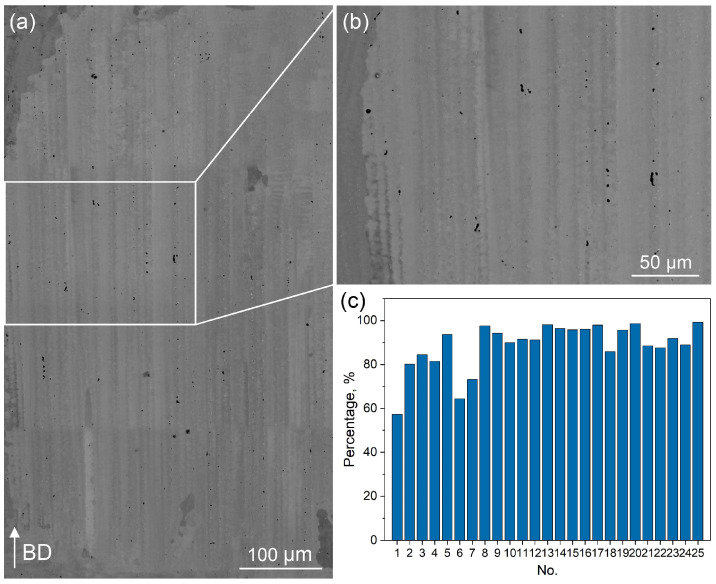
(**a**,**b**) SEM BSE images showing well-aligned, rounded voids (micro-shrinkages) between the dendrite trunks; (**c**) the percentage of voids with an average diameter of less than 5 μm across all 25 samples.

An exhaustive examination of the voids across all 25 samples was conducted, with the results presented in Figure 6. Figure 6a,b are representative SEM BSE images taken from sample No. 20, illustrating rounded voids that are neatly aligned along the direction of columnar grain growth. Mirco-shrinkage voids measuring less than 5 μm in diameter were meticulously tallied in each sample, and the statistical outcomes are summarized in Figure 6c. It is observed that the majority of micro-shrinkage voids account for more than 80% of the total void population. Furthermore, in samples subjected to higher laser power settings, this proportion can even exceed 90%. As described in previous studies [32], these voids typically arise from dendritic secondary arms that bridge, preventing the liquid from reaching the isolated channels and compensating for volume contraction during solidification.

The experimental findings demonstrate that laser power constitutes the predominant determinant of defect formation in additively manufactured superalloys, with scanning velocity exhibiting a secondary influence. Concomitantly, an insufficient powder feed rate was identified as a contributory factor exacerbating defect generation. Based on these correlations, an optimized processing window was established for the alloy: a laser power range of 600~700 W, a linear energy density range of 60~90 J/mm, a relatively high powder feeding rate of 0.7 rpm and a hatching space of 0.75 mm.

### 3.3. Cracking Mechanism of Ni-Based Superalloy

Through a thorough analysis, we established correlations between the process parameters involved in laser AM of Ni-based superalloy and the occurrence of defects, focusing particularly on laser power, scanning speed and linear energy density. However, the exact mechanisms through which these parameters influence cracking remain not completely clear; thus, further research into the microstructure and characteristics of the cracks is essential.

**Figure 7:**
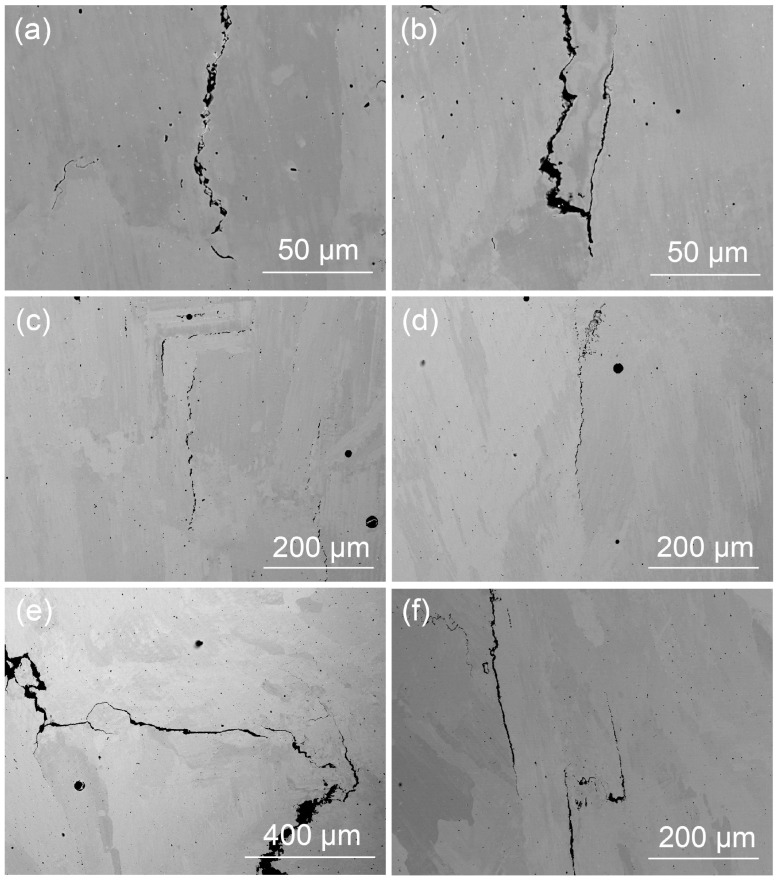
SEM BSE images illustrating the morphology of the cracks in typical Ni-based superalloy samples produced with different linear energy densities: (**a**,**b**) sample No. 12; (**c**,**d**) sample No. 9; (**e**,**f**) sample No. 20.

Figure 7 presents SEM BSE images of samples that were fabricated with varying linear energy densities: sample No. 12 with a low LED value of 45 J/mm, sample No. 9 with a moderate LED value of 80 J/mm and sample No. 20 with a high LED value of 108 J/mm, all captured in the cross-sectional direction of the build. In sample Nos. 12 and 9, cracks were predominantly observed in the inter-dendritic regions, associated with secondary dendrite arms indicative of solidification cracking, as referenced in [36] and depicted in Figure 7a,d. However, in sample No. 20, solid-state cracking was evident, characterized by long, straight cracks with distinct sharp kinks [7], as shown in Figure 7e,f. This can be attributed to the fact that the increased linear energy density results in a higher heat input within the heat-affected zone (HAZ) of the primary cladding layer, which, in turn, intensifies thermal stress and promotes solid-state cracking [7].

**Figure 8:**
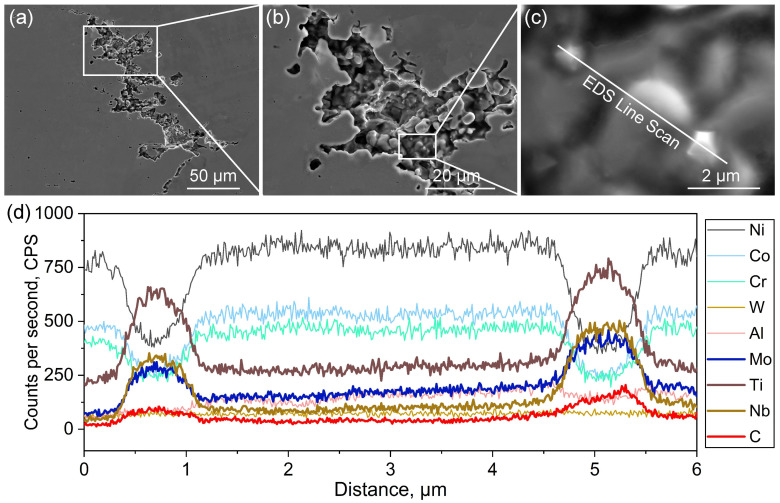
SEM and EDS analysis of the cracks in sample No. 9: SEM images showing (**a**,**b**) the microstructure of solidification cracking and (**c**) the location of an EDS line scan; (**d**) the extracted concentration profiles from the white line in (**c**).

The area of dendrite exposure in sample No. 9, where cracks were notably observed, was specifically selected to facilitate the observation and analysis of solidification cracking, as depicted in Figure 8a. It is noted that numerous secondary dendrites and fine particles were present on the primary dendrite arms (Figure 8b,c). EDS line scans were subsequently conducted across these particles to facilitate a more distinct observation of the elemental distributions. Figure 8d shows the concentration profiles extracted from the location highlighted by a white line in Figure 8c. The presence of enriched C, Ti, Mo and Nb elements in the two brighter particles was confirmed, providing further evidence that these particles are, indeed, MC carbides. During the final stages of solidification (*f*_s_ = 0.9~0.99), the existence of high-melting-point carbides dispersed among dendrites can impede the supply of liquid phase to the mushy zone. This consequently results in micro-shrinkages and triggers the initiation of solidification cracking [49,50].

**Figure 9:**
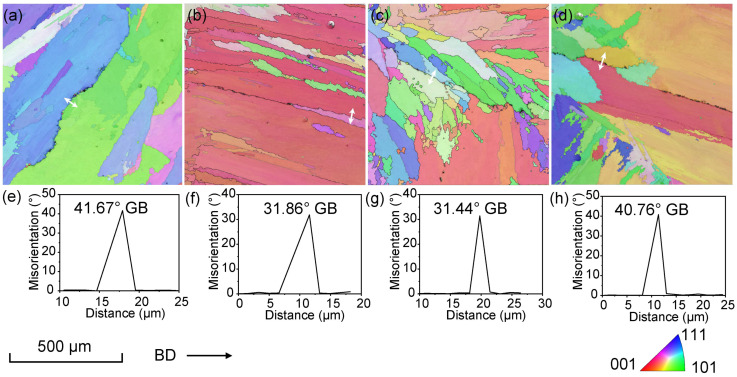
EBSD IPF maps and GB misorientations of cracks in typical Ni-based superalloy samples produced with different linear energy densities: (**a**,**e**) sample No. 16; (**b**,**f**) sample No. 12; (**c**,**g**) sample No. 18; (**d**,**h**) sample No. 20. The white arrows in (**a**–**d**) indicate cracked grain boundaries.

EBSD was utilized to investigate the relationship between the cracks and the misorientation angles of grain boundaries (GBs). The analysis included four typical cracks found in sample Nos. 16, 12, 18 and 20 that were produced with LED values of 42.85 J/mm, 45 J/mm, 70 J/mm and 108 J/mm, respectively. Figure 9 displays a series of inverse pole figure (IPF) maps taken in the vicinity of cracks along the build direction. The insets within each map illustrate the grain-boundary angles at the crack locations, as indicated by white arrows in the corresponding EBSD GBs images. It is observed that cracks predominantly form at GB angles within the range of 31~42°, consistent with the findings reported by Guo [36] that cracks tend to form preferentially in a specific GB misorientation range of 25~45°. GB angles that exhibit a propensity for crack formation are categorized as crack-susceptible high-angle grain boundaries (S-HAGBs).

**Figure 10:**
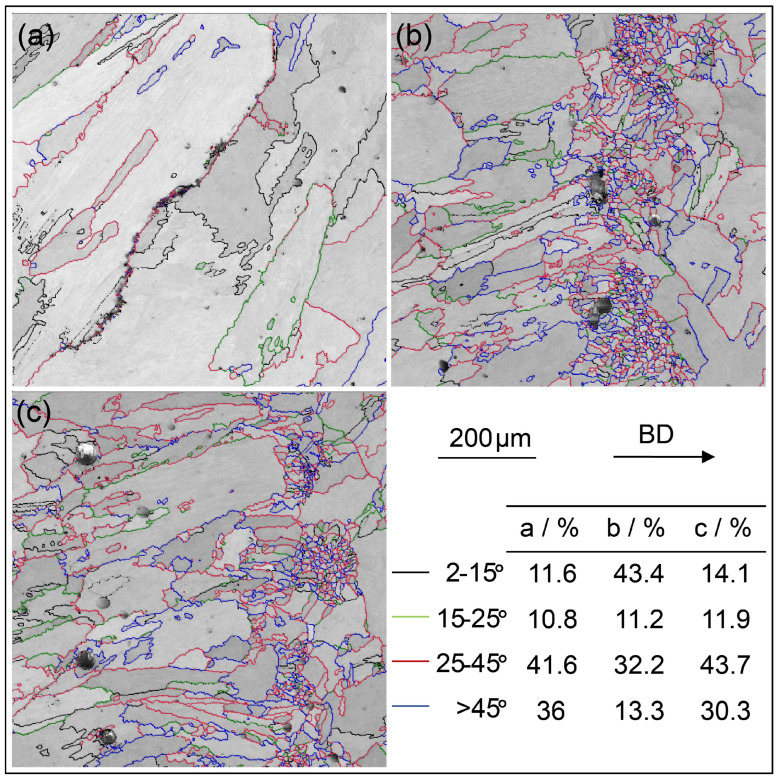
EBSD BC maps showing the GBs in sample No. 16: (**a**) cracked area; (**b**,**c**) uncracked areas.

A detailed EBSD analysis was performed on sample No. 16, and the resulting EBSD band contrast (BC) maps are presented in Figure 10. It is noteworthy that S-HAGBs are observed in both the cracked and uncracked regions. In the uncracked area, a considerable number of very fine grains are detected. As demonstrated by Xu et al. [50], grain refinement can effectively reduce cracking during AM processes. Enhanced crystal nucleation occurs at HAGBs due to the lower energy density, which results in decreased heat input and slower grain growth rates. This promotes the formation of fine grains at stress-induced HAGBs, which can effectively alleviate cracking.

## 4. Conclusions

The influence of critical process parameters on defect formation mechanisms during additive manufacturing (AM) of a novel Ni-based superalloy was comprehensively analyzed. The established correlations between defect generation and processing conditions enable the determination of an optimized parameter window to minimize microstructural imperfections in AM-fabricated Ni-based superalloy components. Based on the findings of the present study, the following conclusions can be drawn:

1. Defect quantity maps for the Ni-based superalloy were created by using key AM process parameters of laser power, scanning speed and linear energy density.
2. As the laser power increases, the void content in the Ni-based superalloy tends to decrease, whereas the crack count and length densities exhibit an increase. An optimal laser power range of 600~700 W was identified as yielding comparatively low levels of both void contents and crack densities.
3. High crack densities were observed in samples produced using a low powder feeding rate of 0.4 rpm. A relatively higher powder feeding rate is recommended to mitigate voids and cracking within alloy samples.
4. The presence of fine MC carbides dispersed among the dendrites was found to cause micro-shrinkages and trigger the initiation of solidification cracking.
5. Grain-boundary misorientations within the range of 31~42° (defined as S-HAGBs) exhibit a high propensity for crack formation, suggesting that S-HAGBs are a critical factor affecting cracking susceptibility.

## Figures and Tables

**Table 1:** The main chemical compositions of the Ni-based superalloy powders (wt.%).

Element	Ni	Co	Cr	Mo + W	Nb	Al + Ti	Hf	C + B + Zr	Si
wt.%	Bal.	25.75	13.00	7.94	0.93	7.1	0.19	0.098	0.048

**Table 2:** L-DED parameters for the Ni-based superalloy.

Condition No.	Power (W)	Scanning Speed (mm/min)	Powder Feeding Rate (rpm)	Hatching Space (mm)	Linear Energy Density (J/mm)
1	500	400	0.4	0.5	75
2	600	600	0.7	0.7	60
3	700	800	0.5	0.65	52.5
4	800	500	0.8	0.6	96
5	900	700	0.6	0.55	77.14
6	500	500	0.5	0.55	60
7	600	700	0.8	0.5	51.43
8	700	400	0.6	0.7	105
9	800	600	0.4	0.65	80
10	900	800	0.7	0.6	67.5
11	500	600	0.6	0.6	50
12	600	800	0.4	0.55	45
13	700	500	0.7	0.5	84
14	800	700	0.5	0.7	68.57
15	900	400	0.8	0.65	135
16	500	700	0.7	0.65	42.86
17	600	400	0.5	0.6	90
18	700	600	0.8	0.55	70
19	800	800	0.6	0.5	60
20	900	500	0.4	0.7	108
21	500	800	0.8	0.7	37.5
22	600	500	0.6	0.65	72
23	700	700	0.4	0.6	60
24	800	400	0.7	0.55	120
25	900	600	0.5	0.5	90

**Table 3:** Summary of the statistical results for voids and cracks in all 25 bulk samples.

Condition No.	Void Content (%)	Crack Count Density (pieces/mm^2^)	Crack Length Density (mm/mm^2^)
1	1.18	0.60	0.07
2	1.14	0.12	0.01
3	0.80	0.65	0.09
4	0.20	1.13	0.17
5	0.22	1.96	0.28
6	0.75	0.00	0.00
7	0.77	0.00	0.00
8	0.38	1.49	0.34
9	0.56	0.60	0.50
10	0.27	0.77	0.15
11	1.15	0.83	0.10
12	1.14	1.25	0.25
13	0.20	0.60	0.13
14	0.26	0.42	0.04
15	0.41	1.01	0.22
16	1.23	0.77	0.14
17	0.38	0.12	0.00
18	0.59	0.36	0.04
19	0.32	0.54	0.08
20	0.19	2.08	0.55
21	0.65	0.48	0.05
22	0.43	0.60	0.04
23	0.21	1.55	0.33
24	0.13	0.60	0.35
25	0.49	1.96	0.14

## Data Availability

The original contributions presented in this study are included in the article. Further inquiries can be directed to the corresponding authors.
